# Dissemination of Clonal Groups of *Brachyspira hyodysenteriae* amongst Pig Farms in Spain, and Their Relationships to Isolates from Other Countries

**DOI:** 10.1371/journal.pone.0039082

**Published:** 2012-06-19

**Authors:** Jesús Osorio, Ana Carvajal, Germán Naharro, Tom La, Nyree D. Phillips, Pedro Rubio, David J. Hampson

**Affiliations:** 1 Animal Health Department, Faculty of Veterinary Science, University of León, León, Spain; 2 School of Veterinary and Biomedical Science, Murdoch University, Murdoch, Western Australia, Australia; University College Dublin, Ireland

## Abstract

**Background:**

Swine dysentery (SD) is a widespread diarrhoeal disease of pigs caused by infection of the large intestine with the anaerobic intestinal spirochaete *Brachyspira hyodysenteriae*. Understanding the dynamics of SD, and hence being able to develop more effective measures to counter its spread, depends on the ability to characterise *B*. *hyodysenteriae* variants and trace relationships of epidemic strains.

**Methodology/Principal Findings:**

A collection of 51 Spanish and 1 Portuguese *B. hyodysenteriae* isolates was examined using a multilocus sequence typing (MLST) scheme based on the sequences of seven conserved genomic loci. The isolates were allocated to 10 sequence types (STs) in three major groups of descent. Isolates in four of the STs were widely distributed in farms around Spain. One farm was infected with isolates from more than one ST. Sequence data obtained from PubMLST for 111 other *B. hyodysenteriae* strains from other countries then were included in the analysis. Two of the predominant STs that were found in Spain also were present in other European countries. The 73 STs were arranged in eleven clonal complexes (Cc) containing between 2 and 26 isolates. A population snapshot based on amino acid types (AATs) placed 75% of the isolates from 32 of the 48 AATs into one major cluster. The founder type AAT9 included 22 isolates from 10 STs that were recovered in Spain, Australia, Sweden, Germany, Belgium, the UK, Canada, and the USA.

**Conclusions/Significance:**

This MLST scheme provided sufficient resolution power to unambiguously characterise *B. hyodysenteriae* isolates, and can be recommended as a routine typing tool that rapidly enables comparisons of isolates. Using this method it was shown that some of the main genetic lineages of *B. hyodysenteriae* in Spain also occurred in other countries, providing further evidence for international transmission. Finally, analysis of AATs appeared useful for deducing putative ancestral relationships between strains.

## Introduction

Bacteria of the genus *Brachyspira* are anaerobic intestinal spirochaetes that can cause diarrhoea and mortality in pigs and other species. This genus comprises seven officially named species and several provisionally named species. Six of these can be found in the porcine large intestine, and currently three are considered to be enteropathogenic to the pig [1]. The most important is *Brachyspira hyodysenteriae*, the aetiological agent of swine dysentery (SD), a severe mucohaemorrhagic diarrhoeal disease that affects the caecum and colon of pigs primarily during the growing-finishing period [2]. The severity of the disease in a herd may vary depending on the virulence of the strain and the level of herd immunity to *B. hyodysenteriae* [3].

SD occurs worldwide and has a major economic impact in many countries, mainly by causing decreased rates of growth, poor feed conversion, the cost of medication and treatments, and deaths [5]. The presence of the disease also is a potential constraint on the trade and movement of pigs between herds. Spain has major problems with SD, as evidenced by the findings that more than 30% of Spanish farms and 12% of porcine faecal specimens tested were positive for *B. hyodysenteriae* [4].

The ability to understand the detailed epidemiology of SD depends on the availability of reliable strain typing methods, which are needed to help trace routes of transmission between farms and countries. Several molecular typing methods have been used for the analysis of *B*. *hyodysenteriae*, including DNA restriction endonuclease analysis [6], [7], random amplification of polymorphic DNA [8], [9], DNA restriction fragment polymorphism analysis [10], and pulsed field gel electrophoresis (PFGE) [9], [11], [12]. In addition, multilocus enzyme electrophoresis (MLEE) and multiple-locus variable-number tandem-repeat analysis (MLVA) have been used to examine the population structure of *B*. *hyodysenteriae* [13]–[15]. Although MLEE has been useful for differentiating and analysing the relatedness of *B*. *hyodysenteriae* strains, the technique is slow and cumbersome to perform, and hence it is not suitable for routine use. In addition, although MLVA is a rapid and simple technique that is useful for local epidemiological studies, the results can be difficult to compare between laboratories unless capillary electrophoresis is used.

Multilocus sequence typing (MLST) has been developed as an alternative method for analysis of microbial population structure and for discriminating between strains [16], [17]. This approach is based on the analysis of sequences of several loci encoding housekeeping genes, and its use has contributed substantially to the understanding of the global epidemiology of many infectious agents. The purpose of the present study was to analyse Spanish porcine isolates of *B*. *hyodysenteriae* using an MLST system previously developed for *B*. *hyodysenteriae* [18], based on modifications to the preliminary scheme described for the whole *Brachyspira* genus [1]. Sequence data obtained for *B*. *hyodysenteriae* in these previous studies have been stored in PubMLST, an expandable global database on a free-access World-Wide Web site. That sequence database enables international exchange of molecular typing data to produce a powerful resource for global epidemiology of SD [18]. The unambiguous characterization of strains of *B*. *hyodysenteriae* is crucial for addressing questions relating to its epidemiology, population structure and evolutionary biology.

## Materials and Methods

### *Brachyspira Hyodysenteriae* Isolates

**Figure 1:**
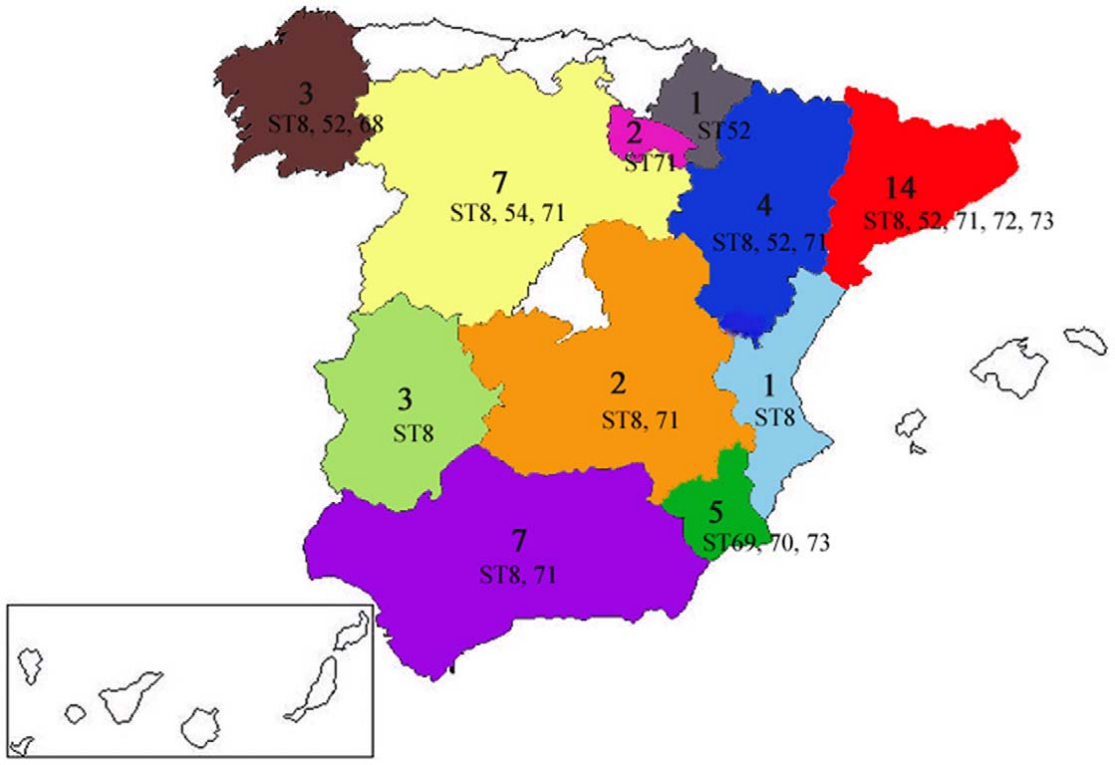
Administrative regions where the Spanish farms were located. For each region, the numbers of isolates are indicated as well as the STs they belong to. The isolate obtained from a Portuguese farm is not shown.

A total of 51 Spanish and 1 Portuguese isolates of *B*. *hyodysenteriae* were obtained as frozen stock from the spirochaete culture collection at the University of León. These were classified as *B*. *hyodysenteriae* according to their phenotype and results of species-specific PCR testing [19]. The isolates were chosen as representatives from throughout Spain, being obtained from 11 of the 15 autonomous regions (73%) and from 21 of the 48 provinces (44%) (Figure 1). The isolates came from 47 farms and were recovered from pigs affected with SD between 2001–2007. One of the isolates (H76) was recovered from a pig experimentally infected with US reference strain B204^R^. The names of the isolates, their origins and their dates of isolation are presented in Table 1. Most of the isolates came from commercial white pigs, but seven were from Iberian pigs, an indigenous rustic breed that is traditionally reared in extensive units. Generally single isolates were used from each farm, but two were analysed for each of five of the farms (Table 1). The isolates were cultured, and DNA was extracted as previously reported [18].

**Table 1:** Information for the 51 Spanish and 1 Portuguese isolates included in the study.

Isolate^a^			MLST genes							Region^b^	Isolation^d^		Province	Origin^c^
	ST	AAT	*pgm*	*adh*	*alp*	*est*	*gdh*	*glp*K	*thi*					
**874**	**8**	**8**	**1**/1	**1**/1	**1**/1	**1**/1	**1**/1	**1**/1	**1**/1	Andalucía	May-01	Málaga		Unknown
**1117**	**8**	**8**	**1**/1	**1**/1	**1**/1	**1**/1	**1**/1	**1**/1	**1**/1	Aragón	Sep-01	Zaragoza		White pig
**E1217**	**8**	**8**	**1**/1	**1**/1	**1**/1	**1**/1	**1**/1	**1**/1	**1**/1	Galicia	Oct-01	Unknown		White pig
**E1231**	**8**	**8**	**1**/1	**1**/1	**1**/1	**1**/1	**1**/1	**1**/1	**1**/1	Cataluña	Oct-01	Gerona		White pig
**1502**	**8**	**8**	**1**/1	**1**/1	**1**/1	**1**/1	**1**/1	**1**/1	**1**/1	Andalucía	Jan-02	Málaga		White pig
**5300**	**8**	**8**	**1**/1	**1**/1	**1**/1	**1**/1	**1**/1	**1**/1	**1**/1	Cataluña	Oct-03	Barcelona		White pig
**E380(J)**	**8**	**8**	**1**/1	**1**/1	**1**/1	**1**/1	**1**/1	**1**/1	**1**/1	Unknown	Jan-01	Unknown		Unknown
**E636(H)**	**8**	**8**	**1**/1	**1**/1	**1**/1	**1**/1	**1**/1	**1**/1	**1**/1	Castilla-León	Dec-04	Soria		Unknown
**E644(H)**	**8**	**8**	**1**/1	**1**/1	**1**/1	**1**/1	**1**/1	**1**/1	**1**/1	Castilla-León	Dec-04	Soria		Unknown
**H5**	**8**	**8**	**1**/1	**1**/1	**1**/1	**1**/1	**1**/1	**1**/1	**1**/1	Extremadura	Jan-07	Badajoz		Iberian pig
**H12(B)**	**8**	**8**	**1**/1	**1**/1	**1**/1	**1**/1	**1**/1	**1**/1	**1**/1	Castilla-León	Feb-07	Salamanca		Iberian pig
**H13(A)**	**8**	**8**	**1**/1	**1**/1	**1**/1	**1**/1	**1**/1	**1**/1	**1**/1	Extremadura	Feb-07	Badajoz		Iberian pig
**H21(A)**	**8**	**8**	**1**/1	**1**/1	**1**/1	**1**/1	**1**/1	**1**/1	**1**/1	Extremadura	Feb-07	Badajoz		Iberian pig
**H24**	**8**	**8**	**1**/1	**1**/1	**1**/1	**1**/1	**1**/1	**1**/1	**1**/1	Cataluña	Feb-07	Lérida		White pig
**H42**	**8**	**8**	**1**/1	**1**/1	**1**/1	**1**/1	**1**/1	**1**/1	**1**/1	Cataluña	Mar-07	Barcelona		Unknown
**H44**	**8**	**8**	**1**/1	**1**/1	**1**/1	**1**/1	**1**/1	**1**/1	**1**/1	C-La Mancha	Apr-07	Toledo		Unknown
**H57**	**8**	**8**	**1**/1	**1**/1	**1**/1	**1**/1	**1**/1	**1**/1	**1**/1	Andalucía	May-07	Sevilla		Unknown
**H67**	**8**	**8**	**1**/1	**1**/1	**1**/1	**1**/1	**1**/1	**1**/1	**1**/1	Castilla-León	Jun-07	Zamora		White pig
**H75(B)**	**8**	**8**	**1**/1	**1**/1	**1**/1	**1**/1	**1**/1	**1**/1	**1**/1	Castilla-León	Jun-07	Salamanca		Iberian pig
**H79**	**8**	**8**	**1**/1	**1**/1	**1**/1	**1**/1	**1**/1	**1**/1	**1**/1	C. Valenciana	Jul-07	Castellón		Unknown
**H81**	**8**	**8**	**1**/1	**1**/1	**1**/1	**1**/1	**1**/1	**1**/1	**1**/1	Andalucía	Jul-07	Córdoba		Iberian pig
**E838**	**52**	**9**	**2**/1	**1**/1	**2**/2	**1**/1	**6**/2	**4**/1	**2**/1	Cataluña	May-01	Barcelona		White pig
**3140**	**52**	**9**	**2**/1	**1**/1	**2**/2	**1**/1	**6**/2	**4**/1	**2**/1	Navarra	Oct-02	Navarra		White pig
**3410**	**52**	**9**	**2**/1	**1**/1	**2**/2	**1**/1	**6**/2	**4**/1	**2**/1	Galicia	Oct-02	Pontevedra		Unknown
**4722**	**52**	**9**	**2**/1	**1**/1	**2**/2	**1**/1	**6**/2	**4**/1	**2**/1	Aragón	Jul-03	Huesca		White pig
**H3**	**52**	**9**	**2**/1	**1**/1	**2**/2	**1**/1	**6**/2	**4**/1	**2**/1	Aragón	Dec-06	Huesca		White pig
**H4**	**52**	**9**	**2**/1	**1**/1	**2**/2	**1**/1	**6**/2	**4**/1	**2**/1	Cataluña	Jan-07	Lérida		White pig
**H9**	**52**	**9**	**2**/1	**1**/1	**2**/2	**1**/1	**6**/2	**4**/1	**2**/1	Cataluña	Jan-07	Barcelona		White pig
**H76**	**54**	**9**	**5**/1	**2**/1	**5**/2	**1**/1	**3**/2	**6**/1	**3**/1	Castilla-León	Jun-07	León		White pig
**H32**	**67**	**44**	6/2	**1**/1	4/2	**1**/1	**7**/2	5/2	**1**/1	PORTUGAL	Mar-07	Alentejo		Unknown
**5074**	**68**	**9**	**2**/1	**1**/1	**2**/2	**1**/1	4/2	**4**/1	**2**/1	Galicia	Oct-03	Unknown		White pig
**H73**	**69**	**8**	**5**/1	**1**/1	**1**/1	**1**/1	**1**/1	**1**/1	**1**/1	Murcia	Jun-07	Murcia		White pig
**H52**	**70**	**45**	4/3	**1**/1	**3**/2	**2**/2	5/2	**3**/1	**1**/1	Murcia	May-07	Murcia		White pig
**H69**	**70**	**45**	4/3	**1**/1	**3**/2	**2**/2	5/2	**3**/1	**1**/1	Murcia	Jun-07	Murcia		White pig
**H19**	**71**	**46**	**5**/1	**1**/1	6/2	**2**/2	8/2	7/1	**1**/1	Cataluña	Feb-07	Barcelona		White pig
**H27**	**71**	**46**	**5**/1	**1**/1	6/2	**2**/2	8/2	7/1	**1**/1	C-La Mancha	Feb-07	Toledo		Unknown
**H31**	**71**	**46**	**5**/1	**1**/1	6/2	**2**/2	8/2	7/1	**1**/1	Castilla-León	Feb-07	Zamora		Iberian pig
**H38**	**71**	**46**	**5**/1	**1**/1	6/2	**2**/2	8/2	7/1	**1**/1	Andalucía	Mar-07	Almería		Unknown
**H46**	**71**	**46**	**5**/1	**1**/1	6/2	**2**/2	8/2	7/1	**1**/1	Aragón	Apr-07	Zaragoza		White pig
**H65**	**71**	**46**	**5**/1	**1**/1	6/2	**2**/2	8/2	7/1	**1**/1	Cataluña	Jun-07	Barcelona		White pig
**Ex81**	**71**	**46**	**5**/1	**1**/1	6/2	**2**/2	8/2	7/1	**1**/1	Cataluña	May-07	Barcelona		White pig
**E377(J)**	**71**	**46**	**5**/1	**1**/1	6/2	**2**/2	8/2	7/1	**1**/1	Unknown	Jan-01	Unknown		Unknown
**E605(I)**	**71**	**46**	**5**/1	**1**/1	6/2	**2**/2	8/2	7/1	**1**/1	La Rioja	Feb-01	La Rioja		White pig
**1002(I)**	**71**	**46**	**5**/1	**1**/1	6/2	**2**/2	8/2	7/1	**1**/1	La Rioja	Jul-01	La Rioja		White pig
**4889**	**71**	**46**	**5**/1	**1**/1	6/2	**2**/2	8/2	7/1	**1**/1	Andalucía	Sep-03	Málaga		Unknown
**5861**	**71**	**46**	**5**/1	**1**/1	6/2	**2**/2	8/2	7/1	**1**/1	Andalucía	Feb-04	Huelva		Unknown
**H15**	**72**	**47**	3/4	**1**/1	**2**/2	**1**/1	**6**/2	**4**/1	**2**/1	Cataluña	Feb-07	Barcelona		White pig
**H1**	**73**	**48**	6/2	**1**/1	**3**/2	**1**/1	**2**/1	**2**/1	**1**/1	Cataluña	Dec-06	Lérida		White pig
**H2**	**73**	**48**	6/2	**1**/1	**3**/2	**1**/1	**2**/1	**2**/1	**1**/1	Cataluña	Dec-06	Lérida		White pig
**H34**	**73**	**48**	6/2	**1**/1	**3**/2	**1**/1	**2**/1	**2**/1	**1**/1	Murcia	Mar-07	Murcia		White pig
**H40**	**73**	**48**	6/2	**1**/1	**3**/2	**1**/1	**2**/1	**2**/1	**1**/1	Murcia	Mar-07	Murcia		White pig
**H71**	**73**	**48**	6/2	**1**/1	**3**/2	**1**/1	**2**/1	**2**/1	**1**/1	Cataluña	Jun-07	Lérida		White pig
**Total**	**10**	**7**	**6/4**	**2/1**	**6/2**	**2/2**	**8/2**	**7/2**	**3/1**					

*Brachyspira hyodysenteriae* isolate names, date of isolation and origin are indicated when these were available. Sequence type (ST) and amino acid type (AAT) in MLST, and their allelic profile (nucleotide/amino acid) are also shown.

a Isolates from the same farm are marked with letters A, B, J, H and I in brackets.

b For most of the isolates the Spanish administrative region is specified. Abbreviations used for two regions are: C. Valenciana, Comunidad Valenciana; C-La Mancha, Castilla-La Mancha.

c Most of the isolates were recovered from white pigs, but some were obtained from Iberian pigs.

d The month-year of isolation is marked.

Isolate H76 was recovered from a pig experimentally infected with US reference strain B204^R^.

Numbers in bold in the allelic profiles correspond to sequences previously described [1], [18].

Data for 111 *B*. *hyodysenteriae* isolates that had been previously analysed [18] were obtained from PubMLST (http://pubmlst.org/bhyodysenteriae/) and were included in the final global analysis with the Spanish isolates. Together this represented a total population of 163 isolates recovered over three decades from Australia (50.3%), Europe (43.6%) and North America (6.1%). Specifically the isolates originated from Australia (n = 82), Spain (n = 51), Sweden (n = 10), the USA (n = 7), Canada (n = 3), the UK (n = 5), Germany (n = 3), Portugal (n = 1) and Belgium (n = 1). Included were *B*. *hyodysenteriae* reference strains B204^R^ (ATCC 31212), B234^R^ (ATCC 31287) and WA1^R^ (ATCC 49526), and the type strain B78^T^ (ATCC 27164) [18]. Most of the isolates were recovered from commercial pigs (n = 152; 93.3%) but six were from feral pigs, two from mallards, one from a rhea and one from a mouse (Table S1).

### Multilocus Sequence Typing

Seven MLST loci previously described for use with *B*. *hyodysenteriae* [18] were used for this study. These were the genes encoding alcohol dehydrogenase (*adh*), alkaline phosphatase (*alp*), esterase (*est*), glutamate dehydrogenase (*gdh*), glucose kinase (*glp*K), phosphoglucomutase (*pgm*), and acetyl-CoA acetyltransferase (*thi*). The primers and PCR conditions used were as previously described [18].

PCR and sequencing were performed at Murdoch University (Perth, Australia). PCRs were performed in 60 µl reaction mixtures with *Taq* DNA polymerase (Invitrogen, Carlsbad, CA, USA). Each PCR reaction set included a positive control represented by *B*. *hyodysenteriae* strain WA1^R^ and a negative control consisting of ultrapure water. The PCR conditions were 95°C for 2 min, followed by 33 cycles at 95°C for 30 sec, 50°C for 15 sec, 72°C for 1 min, followed by an extension period of 5 min at 72°C before cooling to 10°C. The PCR products were purified with the AxyPrep™ PCR Clean-up Kit according to the manufacturer’s instructions (Axygen Scientific, Inc., Union City, CA, USA).

The purified PCR products were sequenced using the BigDye Terminator v3.1 Cycle Sequencing Kit (Applied Biosystems, Foster City, CA, USA) according to the manufacturer’s instructions, using the same primers. Sequencing was performed using the 373A sequencing system (Applied Biosystems). Sequence results were analysed and assembled using the ContigExpress component of VectorNTI Advanced 10 (Invitrogen).

To ensure that the sense strand was being used for the analysis, the sequences for each locus were aligned with the original *B*. *hyodysenteriae* strain WA1^R^ sequence [18], using ClustalW (from EMBL-EBI, European Bioinformatics Institute [http://www.ebi.ac.uk/clustalw/]). The aligned loci sequences were trimmed using GeneDoc [20] and the length of each allele used for the subsequent MLST analysis was as previously described [18]. The DNA sequences also were translated to predicted amino acid sequences using BioEdit version 7.0.9.0 [21].

### Data Analysis

The analysis was conducted initially on the data generated from the Spanish and Portuguese isolates, and subsequently on the combined data from these isolates and the previously available sequences for each locus of the other 111 *B*. *hyodysenteriae* isolates [18]. The alignments for each of the loci were used to identify isolate sequences that were identical. Each unique nucleotide sequence was assigned a unique allele number. The allelic profile for each isolate was determined and consisted of a line listing the allele number for each locus in turn. Isolates were assigned a sequence type (ST) according to their allelic profiles. They were considered genetically identical and hence of the same ST if they were identical at all seven loci. Unique amino acid types (AATs) predicted from the sequences also were recorded. An MLST dendrogram was constructed from the data matrix of allelic mismatches using START2 [22], applying the unweighted pair group method with averages (UPGMA). Isolates were grouped into clonal complexes (Cc) by the BURST algorithm using the eBURST v3 program [23]. A population snapshot was obtained by setting the group definition to 0/7, and assigning a zero for loci without sequence data. The same analysis was used for the AATs. The degree of linkage disequilibrium in the Spanish population and in the whole population was estimated by calculating the Index of Association (I*_A_*) for the isolates and the STs separately [24], using the START2 program. A Diversity Index (DI) based on Simpson’s index of diversity was calculated for the results, as previously described [25].

Allele sequences for each ST also were concatenated using Geneious Pro version 3.8.5 (http://www.geneious.com/) in the gene order *adh*, *pgm*, *est*, *glp*K, *gdh*, *thi* and *alp* used previously [1], [26]. Nucleic acid and deduced amino acid sequences were concatenated in the same order. The concatenated sequences were aligned using ClustalW2 [27] and converted to the MEGA format [28]. Dendrograms depicting relationships for the aligned nucleic acid and amino acid sequences were constructed using the UPGMA method in MEGA version 4.0. The maximum likelihood model was used for the nucleic acid sequences and the Poisson correction model for the amino acid sequences, both with 1000 bootstrap replicates. An unrooted radiation tree was also constructed to help visualise relationships between isolates (not shown). Sequence type designations used in the concatenated trees were the same as in the consensus trees.

## Results

### Spanish and Portuguese Isolates

All genes had excellent sequence quality and did not have deletions or insertions. The 52 isolates were divided into 10 STs and 7 AATs (Table 1). The corresponding allele numbers assigned for all the STs are shown in table 1 and these raw sequences are recorded in the PubMLST site. Allelic frequency over the seven loci ranged from two (for *adh* and *est*) to eight (for *gdh*), with a mean of 4.9. Seven of the 10 STs were newly described and included 44.2% of the isolates (n = 23; ST67-ST73), whilst five of the seven AATs were newly described and included 40.4% of the isolates (n = 21; AAT3-AAT7). Isolate H76 had the same ST and AAT as reference strain B204^R^ (ST54 and AAT9).

The maximum nucleotide difference between the isolates involved a total of 21 nucleotide substitutions over 4071 bp of the sequenced gene fragments (0.5%). The alleles for *adh*, *est* and *thi* found in all the Spanish isolates had previously been deposited at the PubMLST site. One allele for *alp*, two for *glp*K and three for both *pgm* and *gdh* were newly described. At the amino acid level, one variant for *glp*K and three for *pgm* were new. One of the seven newly described STs (ST69) consisted of seven alleles that previously had been described. The rest of the new STs had at least one allele that had not previously been described.

Based on the number of strains tested the population had an I*_A_* value of 0.786, whilst based on the number of STs the I*_A_* was 0.990. Significant linkage disequilibrium was found in both analyses (*P* = 0.001, *P* = 0.002).

**Figure 2:**
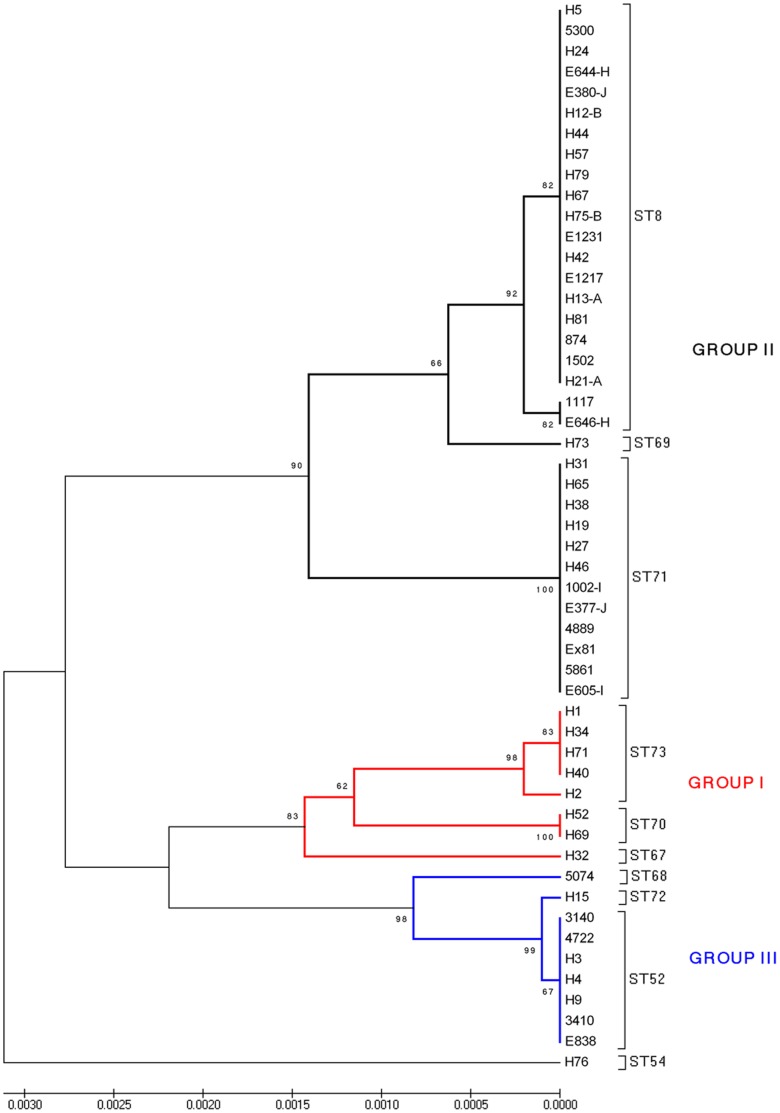
UPGMA dendrogram depicting genetic similarity of the 51 Spanish and one Portuguese *B. hyodysenteriae* isolates used in the study. The tree was constructed using concatenated DNA sequences obtained from the seven MLST loci (*pgm*, *adh*, *alp*, *est*, *glp*K, *gdh* and *thi*: 4,071 nucleotides). Bootstrap values are shown for stable nodes. The scale bar shows the distance equivalent to three substitutions per 1,000 nucleotide positions. The three major groups of descent are marked in different colours. ST8, ST52 and ST54 have been previously described [1], [18].

A dendrogram showing the relative relationships of the 10 STs described for the Spanish and Portuguese isolates is shown as Figure 2. Considering bootstrap values greater than 80%, the isolates were allocated to three major groups of descent. STs 67, 70 and 73 (comprising 8 isolates) belonged to group I, STs 8, 69 and 71 (34 isolates) were included in group II, and STs 52, 68 and 72 (nine isolates) formed group III. ST54 (one isolate) was the most genetically distinct ST, whilst ST52 and ST72 had only one substitution in the *pgm* allele. Five singletons were identified (STs 54, 67, 68, 69 and 72), including one represented by the Portuguese isolate H32. The isolates were moderately heterogeneous (DI value  = 0.768). Overall the number of isolates in an ST varied from 1 to 21 (ST8), and the number of isolates in an AAT varied from 1 to 22 (AAT1) (Tables 1 and 2).

**Table 2:** Information for the 10 STs containing Spanish and Portuguese isolates described in the study.

	N° Isolates	% population	N°Farms	N° Autonomous regions	N° Years
**ST8** *	21	41.4	18	8	5
**ST71**	12	23.1	11	6	4
**ST52** *	7	13.4	7	4	5
**ST73**	5	9.6	5	2	2
**ST70**	2	3.8	2	1	1
**ST54**	1	1.9	1	1	1
**ST67**	1	1.9	1	1	1
**ST68**	1	1.9	1	1	1
**ST69**	1	1.9	1	1	1
**ST72**	1	1.9	1	1	1

* STs that also contained isolates from other countries.

Isolates from four of the 10 STs (ST8, ST52, ST71 and ST73, which included 87% of the isolates) were distributed all around the country (Figure 1). Two isolates from the same farm (J) had different allelic profiles, whilst in four other cases pairs of isolates from the same farms had the same STs. In several cases, isolates recovered from Iberian pigs and white pigs showed genetically identical profiles (ST8 and ST71). Three other STs included more than one isolate (ST52, ST70 and ST73), and all of them were recovered from white pigs suffering from SD.

### Global Population Analysis

When considering the global population of 163 isolates and reference strains, the number of alleles per locus ranged from 9 (*adh*) to 21 (*gdh*), with a mean of 16.9. This implies that the number of different allelic profiles that this scheme can resolve (between 9^7^ and 21^7^) is very high. Consequently, it is unlikely that unrelated isolates exhibit the same allelic profile by chance. In total there were 73 STs and 48 AATs. The number of isolates in an ST varied from 1 to 22, while the number in an AAT varied from 1 to 23. Based on the number of isolates the whole population had an Index of Association (I*_A_*) value of 1.050, whilst based on the number of STs the I*_A_* was 0.175. Significant linkage disequilibrium was found in both analyses (*P* = 0.001). The diversity for this whole population was high, with a DI value of 0.965.

**Figure 3:**
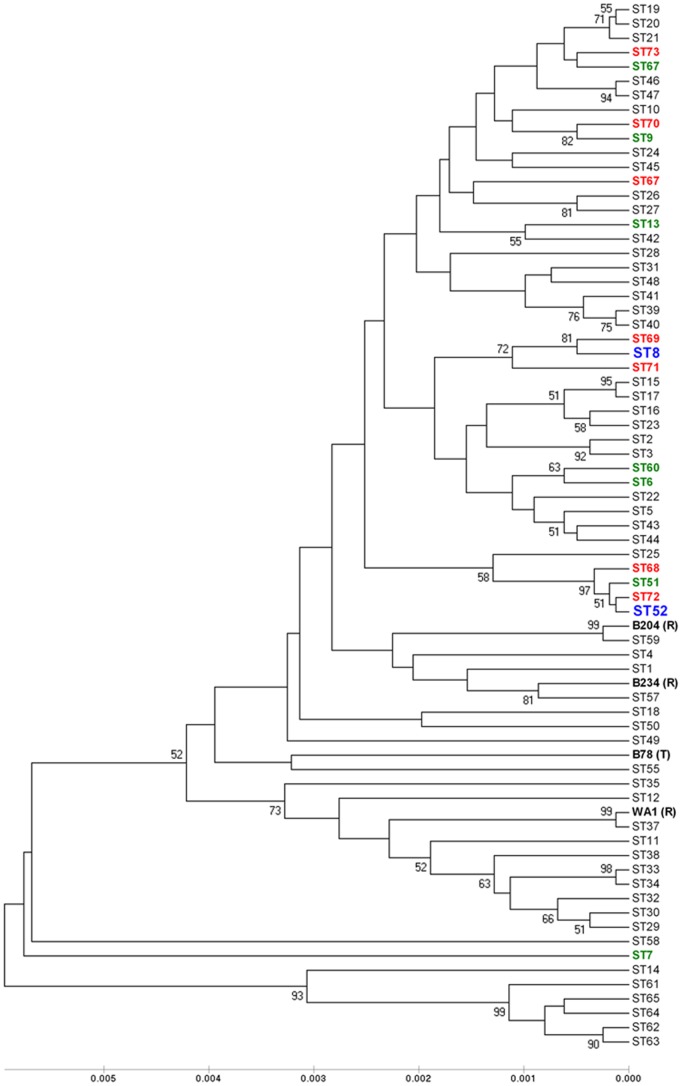
UPGMA dendrogram based on concatenated nucleotide sequences from the seven loci from a population of 163 *B*. *hyodysenteriae* isolates. Bootstrap values greater than 50 are shown in the nodes. The length on the scale indicates a distance of 4 substitutions in the sequence. There was a total of 73 STs, and the seven new STs described for Spanish isolates are indicated in red. The other three STs corresponded to two previously described STs (in blue), and one shared with B204^R^. The green colour indicates STs containing other European isolates. Type and reference strains are indicated. Branch lengths are proportional to genetic distance.

An UPGMA dendrogram showing the relative relationships of these 73 STs is presented as Figure 3. Information about the names, ST and AAT designations of the isolates is summarized in Table S1. Most of the STs (n = 65, 89%) and 71.8% of all the isolates (n = 117) from the studied population were grouped into one large cluster shown at the top of the dendrogram, with the other eight STs (English and Australian origin) generally comprising single isolates (except ST64) that were arranged separately with a percentage of nucleotide substitutions greater than 1% comparing with the rest of the STs. The small cluster comprising ST61-ST65 contained recent isolates from Western Australia.

The 82 isolates from Australia were divided into 46 STs and represented unique genotypes in the population. Of the ten STs containing the 52 isolates from Spain and Portugal, one (ST54), containing isolate H76 recovered from a pig experimentally infected with US reference strain B204^R^, was shared with this reference strain, and two STs were shared with isolates from other European countries. ST8 included 21 Spanish isolates from 18 farms located in eight different autonomous regions, and tiamulin resistant strain E2 from the UK [29]. These were in the same clonal complex (Cc1) as UK strain P134/99 (ST7; atypical 23S rDNA sequence; [3]) and H73 (Spain; ST69). ST52 included seven Spanish isolates from seven farms located in four different regions, German isolates A5677/96 and T20 (indole negative) and Belgian isolate Be45 [12], [30]. These were in the same Cc as the indole negative German isolate T4 [1]. Representatives of the other STs containing European isolates were not identified in Spain.

The relationship between the 48 AATs described for the whole population of 163 isolates is shown in an UPGMA dendrogram as Figure S1. Most of the AATs (n = 33, 69%) were grouped into a major cluster that included 132 isolates (80.9%). The other two groups did not include any Spanish isolates and involved a total of 30 isolates from Australia and only one of European origin (ST58, UK).

**Figure 4:**
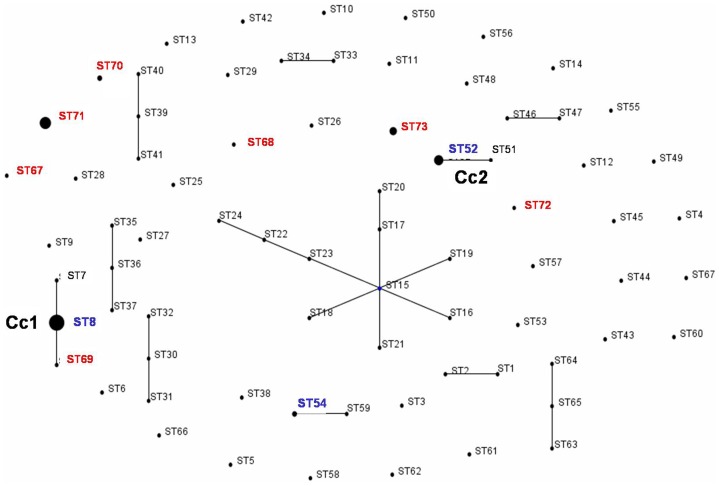
BURST diagram obtained by analysis of nucleotide sequences from seven loci (*pgm*, **D*adh***, *alp*, *est*, *glp*K, *gdh* and *thi*) from a population of 163 *B*. *hyodysenteriae* isolates. The isolates were divided into 73 STs. Isolates that were identical for at least six of the seven loci were included in the same group and are represented by a line between them. Spot diameters are proportional to the number of isolates included in the STs. Position and distance between two points are random and do not provide additional information. The two clonal complexes including Spanish isolates are indicated, with the seven newly described STs marked in red and the other three in blue.

Eleven clonal complexes (Cc) of STs were identified by e-Burst analysis over the 73 STs obtained from the 163 *B*. *hyodysenteriae* isolates (Figure 4). The Ccs contained between 2 and 10 STs, and between 2 and 26 strains. In most cases the Ccs were made up of strains just from Australia. Cc1 contained isolates from Spain and the UK and Cc2 contained isolates from Spain, Belgium and Germany, and hence were likely to be epidemiologically linked (even though the strains came from farms in different countries isolated over two decades). Cc2 contained three strains from Germany and one from Belgium, all of which had the unusual phenotype of being indole negative, and which also were considered to be related based on their PFGE patterns [12].

**Figure 5:**
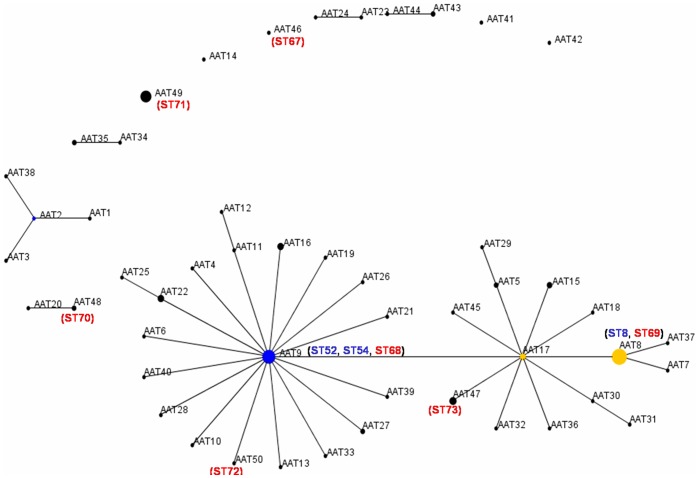
BURST diagram obtained by analysis of translated amino acid sequences from seven loci (*pgm*, *adh*, *alp*, *est*, *glp*K, *gdh* and *thi*) from a population of 163 *B*. *hyodysenteriae* isolates. The isolates were divided into 48 AATs. Isolates that were identical for at least six of the seven loci under study were included in the same group and are represented by a line between them. Spot diameters are proportional to the number of isolates included in the AAT. Position and distance between two points are random and do not provide additional information. The position of the Spanish AATs is indicated in brackets except those forming part of the phenotypes AAT8 and AAT9. The AATs containing isolates from the seven newly described STs are indicated with the number of the ST in red, and the three STs that were previously described are marked in blue.

A population snapshot obtained by using AATs rather than STs is shown in Figure 5. Thirty-two (66.7%) of the AATs were contained in one major cluster, which was made up of two linked sub-clusters. The larger sub-cluster had AAT9 as the founder member, and the other sub-cluster had AAT17 at its centre. This major cluster contained 122 of the strains that were analysed (75.3%). There were four other clusters containing two AATs, one containing four AATs, and each of the other four AATs were separate (one included only Spanish isolates and the other included the Portuguese isolate). Thirty-six (69.2%) of the Spanish isolates were grouped in the major cluster, including isolates from 10 of the 15 autonomous regions (66.6%) and from 18 of the 48 provinces (37.5%). The founder AAT9 profile was shared by 22 isolates from 10 STs, comprising nine from Spain (in ST52, ST54 and ST68), five from Australia, two from Sweden (including a strain from a mouse), two from Germany, and one each from Belgium, the UK, Canada and the USA.

## Discussion

This study was undertaken with the aim of using MLST to analyse the diversity and relationships of Spanish isolates of *B*. *hyodysenteriae*, but was also extended to form a more global overview by including previously available MLST data from isolates from other countries and continents. With the larger data set and by undertaking a comparison of allelic differences at each locus, a more detailed analysis of the whole population was possible.

Results of an earlier study using MLEE suggested that *B*. *hyodysenteriae* is a recombinant species, with an epidemic population structure [14]. On the other hand the MLST study of La et al [18] suggested that the population is clonal. In the current work, the I*_A_* values obtained for the Spanish isolates and for the whole MLST data set were significantly different from zero whether or not they were calculated on the number of strains or number of STs, and this result was consistent with the *B*. *hyodysenteriae* population being clonal.

Analysis of a representative collection of Spanish isolates of *B*. *hyodysenteriae* identified a predominant and widely disseminated ST (ST8). This stable clonal group of isolates originated from 18 different farms affected by SD in 13 provinces of eight autonomous regions. The other Spanish isolates were reasonably diverse, with the 52 isolates being divided overall into 10 STs in three major groups of descent. As none of the isolates differed at all loci, and the maximum distance between them only involved a total of 21 nucleotide substitutions over 4071 bp of the sequenced gene fragments, this tends to suggest that the 10 STs diverged relatively recently.

Spanish isolate H76 in ST54 was identical to US reference strain B204^R^, and as H76 was recovered from a pig inoculated with B204^R^ this confirms the consistency of the MLST method. Other than H76, none of the Spanish isolates were identical to the large number of isolates from Australia and North America. On the other hand, two of the three predominant STs present in Spain previously had been described in other European countries [1], [18], and probably have spread as a result of the regular trade of pigs within Europe. The predominant ST identified in Spain (ST8; Figure 2) was shared by a British tiamulin resistant isolate, E2. In a previous study, E2 and six isolates from several farms with outbreaks of SD in the South-East of England showed the same PFGE pattern, indicating that they belonged to a single clone [29]. At least four of the farms were connected through pig movements [31], and five of the isolates were tiamulin resistant. ST8 also was widespread in Spain (Figure 1), and several of the 18 Spanish farms where it was identified had connections. Most of these isolates were recovered from white commercial pigs, but five were from Iberian pigs. Six of these isolates had been checked for susceptibility to tiamulin, and three were resistant and three were susceptible. The extent to which ST8 has spread since 1998 is unknown. Tiamulin is one of the few drugs available for treatment of SD in Spain, as in many other countries, and it is of concern that this clone and other tiamulin-resistant clones are likely to be selected for by tiamulin use, and then transmitted. The other prominent ST (ST52; Figure 2) included seven isolates from seven farms located in four different autonomous regions in northern Spain (Figure 1). This genotype previously was described for two German indole-negative isolates, one of them tiamulin resistant, and isolate Be45 from Belgium [1], [12]. At least three of the Spanish ST52 isolates also were indole-negative [9]. Although ST52 was widespread in Spain, it was not recovered from Iberian pigs. Even though isolates belonging to other European STs were not identified in Spain, this may be because there is limited MLST data available overall. Clearly further studies are needed.

Of the pairs of isolates examined from five of the Spanish farms, only the two isolates from farm J, isolated at the same time, belonged to different STs (ST8 and ST68; Table 1). BURST analysis indicated that these two STs did not belong to the same clonal complex, confirming that genetically diverse isolates were present on the same farm. This is important information as the different strains may vary in their properties, such as antimicrobial resistance, and this could influence the success of control programmes. Similarly, where different strains co-exist on a farm this increases the opportunity for exchange of genetic information between them via the activity of the prophage-like gene transfer agent VSH-1 [32], [33].

It was interesting that ST8 and ST71 contained isolates from both commercial white pigs and Iberian pigs. Movement of stock between these types of farms would be unusual, but the strains could have been transmitted by other means, such as mechanical carriage by migrating birds, rodents, trucks transporting food or pigs, farm personnel, or visitors [5].

At the global level the dendrogram shown as Figure 3, representing all 163 isolates, confirmed previous observations that the species is diverse [13], [14], [18]. There was one larger group of 65 STs, with the rest of the STs at the periphery of the dendrogram containing mainly Australian isolates that were rather distantly related to the European isolates. Presumably these Australian isolates have evolved in isolation, and have not been transmitted to other countries. The major cluster included isolates recovered in different decades and countries, including most of the European isolates.

Analysis of AATs in a population snapshot revealed that the majority (75.3%) of isolates belonged to one major cluster, with AAT9 at its centre (Figure 5). The majority of the Spanish isolates (69.2%) also belonged to that major cluster. The AAT9 phenotype was shared by strains from different countries, including nine isolates from Spain, and even included an isolate from a mallard and one from a mouse. As previously suggested [18], it seems likely that AAT9 represents an ancestral type, with the other AATs (and STs) having arisen from genetic drift associated with mutational or other genetic changes.

Using AATs instead of STs gave a clearer identification of isolates that were closely related, irrespective of their place of isolation. The greater clustering of AATs compared to STs suggests that there is a negative selection on changes in nucleotide sequences that result in amino acids changes that disrupt the function of the housekeeping enzymes. Overall the use of translated amino acid sequences helped to more clearly define the species and its boundaries, whilst organisation of isolates into Cc made the MLST data more amenable to epidemiological analysis.

### Implications

This study has demonstrated that MLST is a powerful method to characterise and compare isolates of *B. hyodysenteriae* from different countries. Using MLST it was possible to show that closely related strains or clonal groups of the spirochaete were present in pig farms throughout Spain, as well as in some other European countries. Accordingly, MLST can be recommended as a routine typing tool for *B. hyodysenteriae* that enables rapid international comparisons of isolates. Analysis of AATs appears to be useful as a means of deducing putative ancestral relationships between strains. Overall this study has contributed to understanding of the dynamics of the *B*. *hyodysenteriae* population, which in turn can help to determine appropriate interventions, including ongoing pathogen surveillance, and inform development of appropriate vaccines and therapeutics.

## Supporting Information

Figure S1 **UPGMA dendrogram based on concatenated amino acid sequences from 7 loci (** ***pgm*** **,** ***adh*** **,** ***alp*** **,** ***est*** **,** ***glp*** **K,** ***gdh*** **and** ***thi*** **; 1,357 amino acids) from a population of 163** ***B*** **.** ***hyodysenteriae*** **isolates and strains.** A total of 48 AATs were identified, and the STs included in each AAT are indicated [18]. The new Spanish STs are indicated in red, the two STs shared with European isolates are marked in blue and the tenth ST is shared with B204^R^. Most of the isolates in the whole population (n = 132, 80.9%) were grouped into a major cluster that included all Spanish isolates (n = 52; 100%), located at the top of the dendrogram. The length of a space on the scale indicates a distance of 1.3 substitutions in the peptide sequence.
(TIF)

Table S1 **Sequence type (ST), amino acid type (AAT) name and origin of the 163** ***B. hyodysenteriae*** **isolates analysed.** The isolates were all from pigs, unless otherwise noted (Råsbäck et al., 2007; La et al., 2009). The new isolates from Spain and Portugal are shown in the shaded rows.
(DOCX)
