# Supplementary figures and images for: Dissemination of Clonal Groups of Brachyspira hyodysenteriae amongst Pig Farms in Spain, and Their Relationships to Isolates from Other Countries
Source: PLoS One. 2012 Jun 19;7(6):e39082. doi: 10.1371/journal.pone.0039082 (PMC3378525; doi:10.1371/journal.pone.0039082)

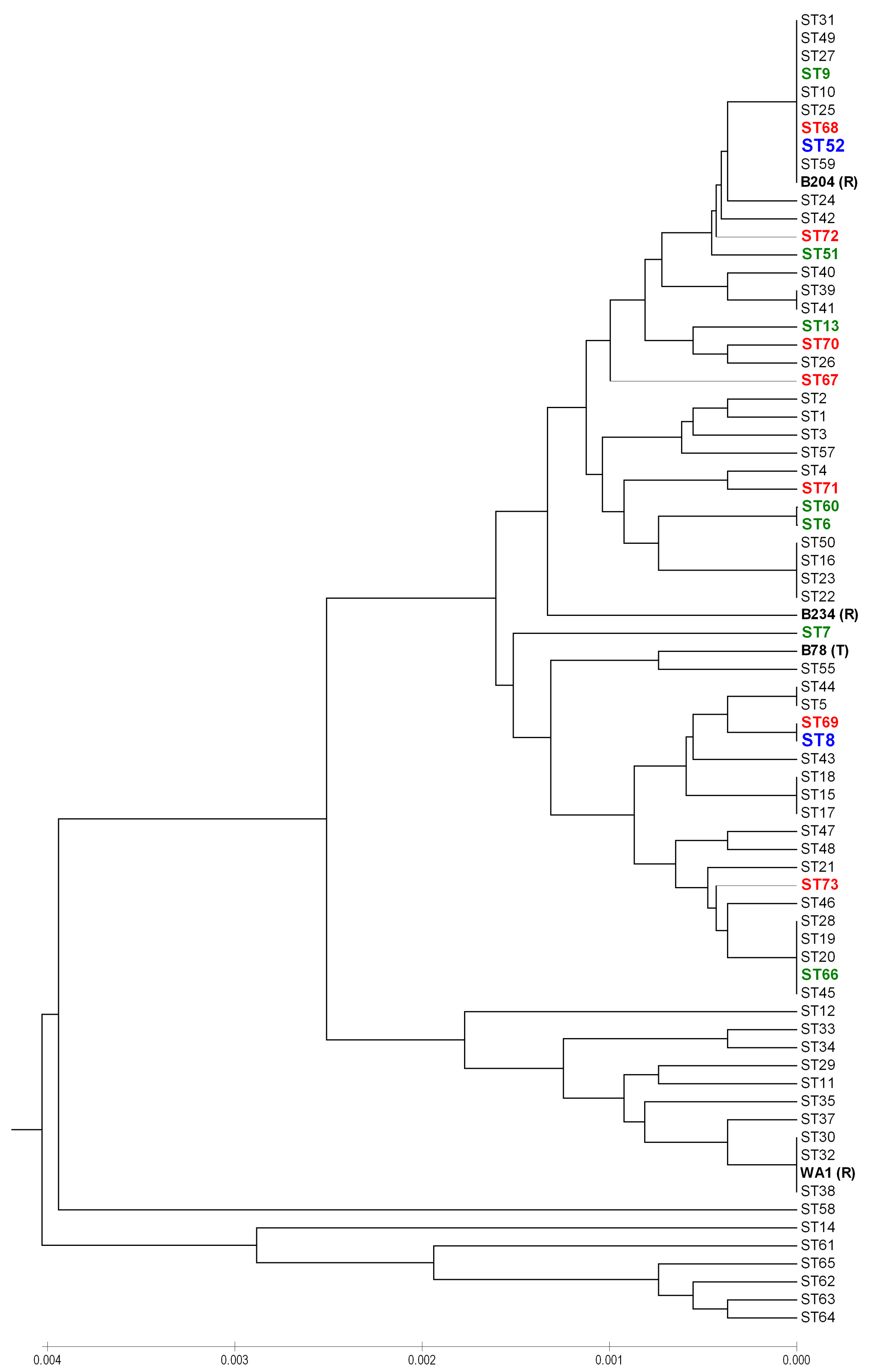

Supplement: Figure S1 — UPGMA dendrogram based on concatenated amino acid sequences from 7 loci ( pgm , adh , alp , est , glp K, gdh and thi ; 1,357 amino acids) from a population of 163 B . hyodysenteriae isolates and strains. A total of 48 AATs were identified, and the STs included in each AAT are indicated [18]. The new Spanish STs are indicated in red, the two STs shared with European isolates are marked in blue and the tenth ST is shared with B204R. Most of the isolates in the whole population (n = 132, 80.9%) were grouped into a major cluster that included all Spanish isolates (n = 52; 100%), located at the top of the dendrogram. The length of a space on the scale indicates a distance of 1.3 substitutions in the peptide sequence. (TIF) [file pone.0039082.s001.tif]
