# Supplementary material for: Dissemination of Clonal Groups of Brachyspira hyodysenteriae amongst Pig Farms in Spain, and Their Relationships to Isolates from Other Countries
Source: PLoS One. 2012 Jun 19;7(6):e39082. doi: 10.1371/journal.pone.0039082 (PMC3378525; doi:10.1371/journal.pone.0039082)
Supplement: Table S1 — Sequence type (ST), amino acid type (AAT) name and origin of the 163 B. hyodysenteriae isolates analysed. The isolates were all from pigs, unless otherwise noted (Råsbäck et al., 2007; La et al., 2009). The new isolates from Spain and Portugal are shown in the shaded rows. (DOCX) [file pone.0039082.s002.docx]

| ST | AAT | Isolate name | Origin |
| --- | --- | --- | --- |
| 1 | 1 | WA46 | Australia, 2007 (Farm A) |
| 2 | 2 | WA41 | Australia, 2007 (Farm A) |
| 2 | 2 | WA52 | Australia, 2007 (Farm A) |
| 2 | 2 | WA53 | Australia, 2007 (Farm A) |
| 2 | 2 | WA56 | Australia, 2007 (Farm A) |
| 2 | 2 | WA57 | Australia, 2007 (Farm A) |
| 2 | 2 | WA42 | Australia, 2007 (Farm A) |
| 3 | 3 | SA12 | Australia, 2007 (Farm C) |
| 4 | 4 | FMV89.3323 | Canada, 1989 |
| 5 | 5 | ACK300/8 | USA, 1970s |
| 6 | 6 | AN3730/96 | Sweden |
| 6 | 6 | AN613/98 | Sweden |
| 6 | 6 | AN2420/97 | Sweden |
| 7 | 7 | P134/99 | UK |
| 8 | 8 | E2 | UK |
| 8 | 8 | 874 | Spain |
| 8 | 8 | 1117 | Spain |
| 8 | 8 | E1217 | Spain |
| 8 | 8 | E1231 | Spain |
| 8 | 8 | 1502 | Spain |
| 8 | 8 | 5300 | Spain |
| 8 | 8 | E380(J) | Spain |
| 8 | 8 | E636(H) | Spain |
| 8 | 8 | E644(H) | Spain |
| 8 | 8 | H5 | Spain |
| 8 | 8 | H12(B) | Spain |
| 8 | 8 | H13(A) | Spain |
| 8 | 8 | H21(A) | Spain |
| 8 | 8 | H24 | Spain |
| 8 | 8 | H42 | Spain |
| 8 | 8 | H44 | Spain |
| 8 | 8 | H57 | Spain |
| 8 | 8 | H67 | Spain |
| 8 | 8 | H75(B) | Spain |
| 8 | 8 | H79 | Spain |
| 8 | 8 | H81 | Spain |
| 9 | 9 | AN360/03 | Sweden |
| 9 | 9 | AN551/03 | Sweden, mouse |
| 10 | 9 | KF9 | UK, 1970s |
| 11 | 11 | Vic36 | Australia, 1991 |
| 12 | 12 | Vic30 | Australia, 1980s |
| 12 | 12 | Vic32 | Australia, 1980s |
| 13 | 13 | AN174/92 | Sweden |
| 13 | 13 | AN1082/90 | Sweden |
| 13 | 13 | AN3379/98 | Sweden |
| 14 | 14 | Vic38 | Australia, 1990 |
| 15 | 15 | NSW13 | Australia, 1990s |
| 15 | 15 | NSW14 | Australia, 1990s (Farm D) D)D) |
| 16 | 16 | SA2 | Australia, 1980s |
| 17 | 15 | Vic4 | Australia, 1980s |
| 18 | 15 | NSW2 | Australia, 1990s |
| 19 | 17 | NSW42 | Australia, 2005 (Farm D) |
| 19 | 17 | NSW45 | Australia, 2005 (Farm D) |
| 19 | 17 | NSW46 | Australia, 2005 (Farm D) |
| 19 | 17 | NSW49 | Australia, 2005 (Farm D) |
| 19 | 17 | NSW27 | Australia, 2004 (Farm D) |
| 19 | 17 | NSW28 | Australia, 2004 (Farm D) |
| 19 | 17 | NSW32 | Australia, 2005 (Farm D) |
| 19 | 17 | WA40 | Australia, 2005 (Farm B) |
| 20 | 17 | Vic24 | Australia, 1988 |
| 20 | 17 | Vic25 | Australia, 1980s |
| 20 | 17 | Vic33 | Australia, 1980s |
| 21 | 18 | NSW44 | Australia, 2005 (Farm D) |
| 21 | 18 | NSW30 | Australia, 2005 (Farm D) |
| 21 | 18 | NSW34 | Australia, 2005 (Farm D) |
| 22 | 16 | Q1 | Australia, 1980s |
| 22 | 16 | Q21 | Australia, 1980s |
| 22 | 16 | Q3 | Australia, 1980s |
| 22 | 16 | Q8 | Australia, 1980s |
| 22 | 16 | Q9 | Australia, 1980s |
| 23 | 16 | SA1 | Australia, 1980s |
| 24 | 19 | B8044 | USA, 1980s |
| 25 | 9 | SA3 | Australia, 1980s |
| 26 | 20 | B6933 | USA, 1980s |
| 27 | 9 | Vic23 | Australia, 1988 |
| 28 | 17 | NSW9 | Australia, 1991 |
| 28 | 17 | Q33a | Australia, 1991 |
| 29 | 21 | Vic35 | Australia, 1980s |
| 30 | 9 | Vic2 | Australia, 1987 |
| 31 | 9 | WA26 | Australia, 1980s |
| 32 | 22 | Q10 | Australia, 1980s |
| 32 | 22 | Q11 | Australia, 1980s |
| 32 | 22 | Q14 | Australia, 1988 |
| 32 | 22 | Vic31 | Australia, 1980s |
| 33 | 23 | NSW1 | Australia, 1990s |
| 34 | 24 | NSW3 | Australia, 1990s |
| 35 | 25 | Q17 | Australia, 1990s |
| 36 | 22 | Q22 | Australia, 1990s |
| 36 | 22 | WA1^R^ | Australia, 1980s |
| 36 | 22 | WA2 | Australia, 1980s |
| 36 | 22 | WA4 | Australia, 1980s |
| 36 | 22 | WA5 | Australia, 1980s |
| 36 | 22 | WA8 | Australia, 1980s |
| 36 | 22 | WA9 | Australia, 1980s |
| 37 | 26 | WA13 | Australia, 1980 |
| 38 | 22 | FP2 | Australia, 2006 |
| 39 | 27 | FP1 | Australia, 2006 |
| 40 | 9 | WA28 | Australia, 1980s |
| 41 | 27 | FP5 | Australia, 2006 |
| 42 | 28 | R301 | USA (Rhea), 1996 |
| 43 | 29 | WA27 | Australia, 1980s |
| 44 | 5 | WA14 | Australia, 1980 |
| 44 | 5 | WA34 | Australia, 1990s |
| 45 | 17 | FP6 | Australia, 2006 |
| 46 | 30 | FP3 | Australia, 2006 |
| 47 | 31 | Q18 | Australia, 1990s |
| 47 | 31 | WA6 | Australia, 1980s |
| 48 | 32 | FP4 | Australia, 2006 |
| 49 | 9 | SA11 | Australia, 2007 (Farm C) |
| 49 | 9 | WA62 | Australia, 2007 (Farm E) |
| 50 | 16 | Q20 | Australia, 1990s |
| 51 | 33 | T4 | Germany |
| 52 | 9 | A5677/96 | Germany |
| 52 | 9 | Be45 | Belgium |
| 52 | 9 | T20 | Germany |
| 52 | 9 | E838 | Spain |
| 52 | 9 | 3140 | Spain |
| 52 | 9 | 3410 | Spain |
| 52 | 9 | 4722 | Spain |
| 52 | 9 | H3 | Spain |
| 52 | 9 | H4 | Spain |
| 52 | 9 | H9 | Spain |
| 53 | 34 | B234^R^ | USA, 1970s |
| 54 | 9 | H76 | Spain |
| 54 | 9 | B204^R^ | USA, 1970s |
| 55 | 36 | FM88.90 | Canada, 1990 |
| 56 | 37 | B78^T^ | USA, 1970s |
| 57 | 38 | P18A | UK, 1970s |
| 58 | 39 | VS1 | UK, 1970s |
| 59 | 9 | B169 | Canada, 1970s |
| 60 | 6 | AN383:2/00 | Sweden, mallard |
| 61 | 41 | WA73 | Australia, 2007 (Farm B) |
| 62 | 42 | WA71 | Australia, 2007 Farm B) |
| 63 | 43 | WA75 | Australia, 2007 (Farm B) |
| 64 | 44 | WA65 | Australia, 2007 (Farm C) |
| 64 | 44 | WA69 | Australia, 2007 (Farm C) |
| 64 | 44 | WA48 | Australia, 2007 (Farm B) |
| 64 | 44 | WA49 | Australia, 2007 (Farm A) |
| 65 | 43 | WA47 | Australia, 2007 (Farm A) |
|  |  |  |  |
| 66 | 17 | AN1409:2/01 | Sweden, mallard |
| 67 | 44 | H32 | Portugal |
| 68 | 9 | 5074 | Spain |
| 69 | 8 | H73 | Spain |
| 70 | 45 | H52 | Spain |
| 70 | 45 | H69 | Spain |
| 71 | 46 | H19 | Spain |
| 71 | 46 | H27 | Spain |
| 71 | 46 | H31 | Spain |
| 71 | 46 | H38 | Spain |
| 71 | 46 | H46 | Spain |
| 71 | 46 | H65 | Spain |
| 71 | 46 | Ex81 | Spain |
| 71 | 46 | E377(J) | Spain |
| 71 | 46 | E605(I) | Spain |
| 71 | 46 | 1002(I) | Spain |
| 71 | 46 | 4889 | Spain |
| 71 | 46 | 5861 | Spain |
| 72 | 47 | H15 | Spain |
| 73 | 48 | H1 | Spain |
| 73 | 48 | H2 | Spain |
| 73 | 48 | H34 | Spain |
| 73 | 48 | H40 | Spain |
| 73 | 48 | H71 | Spain |
